# Loss of caspase-2 accelerates age-dependent alterations in mitochondrial production of reactive oxygen species

**DOI:** 10.1007/s10522-013-9415-x

**Published:** 2013-03-16

**Authors:** Marisa Lopez-Cruzan, Brian Herman

**Affiliations:** Department of Cellular and Structural Biology, School of Medicine, Barshop Institute for the Study of Aging and Longevity, University of Texas Health Science Center at San Antonio, STRF MC 8254, 8403 Floyd Curl Drive, San Antonio, TX 78229-3904 USA

**Keywords:** Caspase-2, Mitochondria, Reactive oxygen species, Hepatocytes, Aging

## Abstract

Mitochondria are known to be a major source and target of oxidative stress. Oxidative stress increases during aging and is suggested to underlie in part the aging process. We have previously documented an increase in endogenous caspase-2 (casp2) activity in hepatocytes obtained from old (28 months) vs. young mice (5 months). More recently, we have shown that casp2 is activated by oxidative stress and is critical for mitochondrial oxidative stress-induced apoptosis. Since casp2 appears integral to mitochondrial oxidative stress-induced apoptosis, in this study we determined whether loss of casp2 altered the production of mitochondrial reactive oxygen radicals (mROS) as a function of age in intact living hepatocytes. To stimulate mitochondrial metabolic activity, we added a mixture of pyruvate and glutamate to hepatocytes while continuously monitoring endogenous mROS production in the presence or absence of rotenone and/or antimycin A. Our data demonstrate that mROS production and neutralization are compromised in hepatocytes of old mice. Interestingly, casp2 deficient hepatocytes from middle age mice (12 months) had similar mROS neutralization kinetics to those of hepatocytes from old WT mice. Rotenone had no effect on mROS metabolism, whereas antimycin A significantly altered mROS production and metabolism in an age-dependent fashion. Our results indicate that: (1) hepatocytes from young and old mice respond differently to dysfunction of the mitochondrial electron transport chain; (2) age-dependent alterations in mROS metabolism are likely regulated by complex III; and (3) absence of casp2 accelerates age-dependent changes in terms of pyruvate/glutamate-induced mROS metabolism.

## Introduction

With aging, there is a general decrease in liver size and thus in hepatocyte number (Thomas et al. 2002). Rat hepatocytes demonstrate reductions in both proliferative capacity and resistance to oxidative stress as a function of aging (Ikeyama et al. 2003). Previous studies in aged liver have reported oxidant stress-induced damage to mitochondria macromolecules (Rabek et al. 2003, Lopez-Torres et al. 2002, Bakala et al. 2003).

We have previously reported that liver of old mice have increased cysteine oxidation (Zhang et al. 2007) that parallels age-related dysfunction of mitochondria and is thought to be a major determinant of this decline in cell function, since these organelles are both the main sources of reactive oxygen species (ROS) and targets for their damaging effects (Lanza and Nair 2012). Age-associated damage to mitochondria is a consequence of increased oxidant production (Calabrese et al. 2011), probably due to changes in the activity of key components of the respiratory chain (Ralph et al. 2011). Recent studies indicate that mitochondria are one of the major sources of cellular ROS and, in turn, are the most adversely affected organelles during aging (Lee and Wei 2012).

Liver shows a progressive increase with age in the activity of caspases that mediate apoptosis induced through the mitochondrial pathway (Zhang et al. 2002). In fact, the extent of liver apoptosis is higher in old mice that have reduced levels of MnSOD than in age-matched WT mice (Kokoszka et al. 2001). In addition, hepatocytes isolated from old rats are more sensitive to oxidant-induced cell death than hepatocytes isolated from young rats (Zhang et al. 2002).

Casp2 is a member of a family of cysteine proteases that are key regulatory components in apoptosis (Bouchier-Hayes and Green 2012). Casp2 has been found to be expressed in many cell types (Krumschnabel et al. 2009), and localized in several subcellular compartments (Susin et al. 1999, Mancini et al. 2000, Guo et al. 2002, Troy and Shelanski 2003). We have recently shown that casp2 is critical for mitochondrial oxidative stress-induced apoptosis because its absence protects cells treated with mitochondrial complex I and III inhibitors, such as rotenone and antimycin A from apoptosis (Lopez-Cruzan et al. 2005, Tiwari et al. 2011). We also have shown that mitochondria contain oxidative stress activatable casp2 (Lopez-Cruzan et al. 2005). Interestingly, we found increased casp2 activity in aged rat livers that is associated with age-dependent increases in oxidative stress (Zhang et al. 2002). Therefore, casp2 may play a role in oxidative stress-induced apoptosis in aged animals. However, little is known about the function of casp2 in vivo. We have recently found that old male *Casp2* ^−*/*−^ mice exhibit several traits commonly observed in premature aging animals, including a 10 % shortened maximum lifespan and severe age-related osteoporosis (Zhang et al. 2007).

Casp2 has been placed as a central player in the mitochondrial pathway of apoptosis (Robertson et al. 2002; Braga et al. 2008; Madesh et al. 2009; Bouchier-Hayes and Green 2012; Jiang et al. 2012). Small interfering RNA (siRNA) to casp2 inhibited expression of casp2, prevented cytochrome c and Smac release from mitochondria and apoptosis after treatment with cytotoxic agents that can potentially generate ROS (e.g. etoposide, cisplatin and UV irradiation) (Lassus et al. 2002).

In this study, we determined whether loss of casp2 impacted mROS production and metabolism during aging in mouse hepatocytes as a model. Our data indicate that loss of casp2 accelerates age-dependent alterations in mROS production and metabolism, likely through complex III and may in part explain the accelerated aging phenotype that we observe in casp2 null mice (Zhang et al. 2007).

## Materials and methods

### Reagents

Rotenone, antimycin A, Pyruvate, Collagenase Type IV, and Glutamate were purchased from Sigma. MitoSOX (510 nm excitation, 580 nm emission) was obtained from Molecular Probes.

### Mice

*Casp2* ^−*/*−^ mice were a gift from Dr. Carol Troy. Only male mice were used in these studies. In the present studies, we used mice at ages 5, 12 and 28 months of age. This represents for 5 month old WT mice 12 % of their maximum lifespan, 73.9 % for 28 month old WT, and 28.7 % and 31.7 % for 12 month old WT and *Casp2* ^−*/*−^ respectively.

### Hepatocyte isolation

Hepatocytes were isolated using the method of Herman et al. 1988, from 5 and 28 month old male WT mice or 12 month old *Casp2* ^−*/*−^ and WT mice. In brief, animals were anesthetized and their livers perfused with basic medium solution containing 0.5 mM EGTA (115 mM NaCl, 5 mM KCl, 1 mM KH2PO4, 25 mM Na-HEPES) followed by a collagenase solution containing 1 mM CaCl2. After washing and dispersing the cells, they were counted. An average of 25 million hepatocytes per liver was obtained.

### Mitochondrial production of ROS

Animals were handled according to the University’s Institutional Animal Care and Use Committee approved regulations, protocols, and standards. Hepatocytes were isolated from 5 to 28 month old WT mice or 12 month old *Casp2* ^−*/*−^ and WT mice. After hepatocytes were isolated as explained above, cells were washed and dispersed, and counted. After determining cell viability (85–95 %), hepatocytes were seeded at 250,000 cells per well in two-well glass chambers and incubated for a minimum of 9 h to a maximum of 20 h. Generation of ROS over time was monitored with MitoSOX as previously described (Ramanujan and Herman 2007). Cells were washed once and incubated with 2.5 μM MitoSOX during 10 min at 37 °C in the dark and diluted with HBSS containing Ca^2+^ and Mg^2+^, followed by two washes with buffer. HEPES was added at a final concentration of 30 mM to avoid fluctuations in pH while the measurements were accomplished. Readings were performed in a confocal inverted LSM510 Zeiss microscope using a 60× oil immersion objective and an argon laser to detect MitoSOX through a 514 nm excitation/560 nm emission channel. Recordings were performed in live cells using 70 scans in a time period of 13–15 min. After the 20th scan, a mixture of pyruvate and glutamate (PG) was added to the chamber to a final concentration of 5 mM. In some experiments, hepatocytes were treated with 20 μM of rotenone, 20 μM antimycin A or 20 μM of a mixture of both and cells were incubated for 30 min. Cells were then washed with PBS and prepared as described above for mROS measurements. Three regions of interest were independently scanned and each experiment was repeated from six to nine times. Data was normalized for basal intensity by first dividing the intensity obtained at each time point by the initial intensity. Then, the best fit curve in nonlinear regression was found and used to normalize each time point. Analysis was performed with GraphPad Prism software, version 5. For specific time points, one-way ANOVA was used to analyze statistical differences, followed by Bonferroni posttests to discern what groups where significant. At least three animals were used for each sampling group. For the untreated experiments, results were pooled together and statistically analyzed. The results provided for hepatocytes treated with mitochondrial complex inhibitors are presented as individual graphs to highlight the similar response pattern.

## Results

**Fig. 1:**
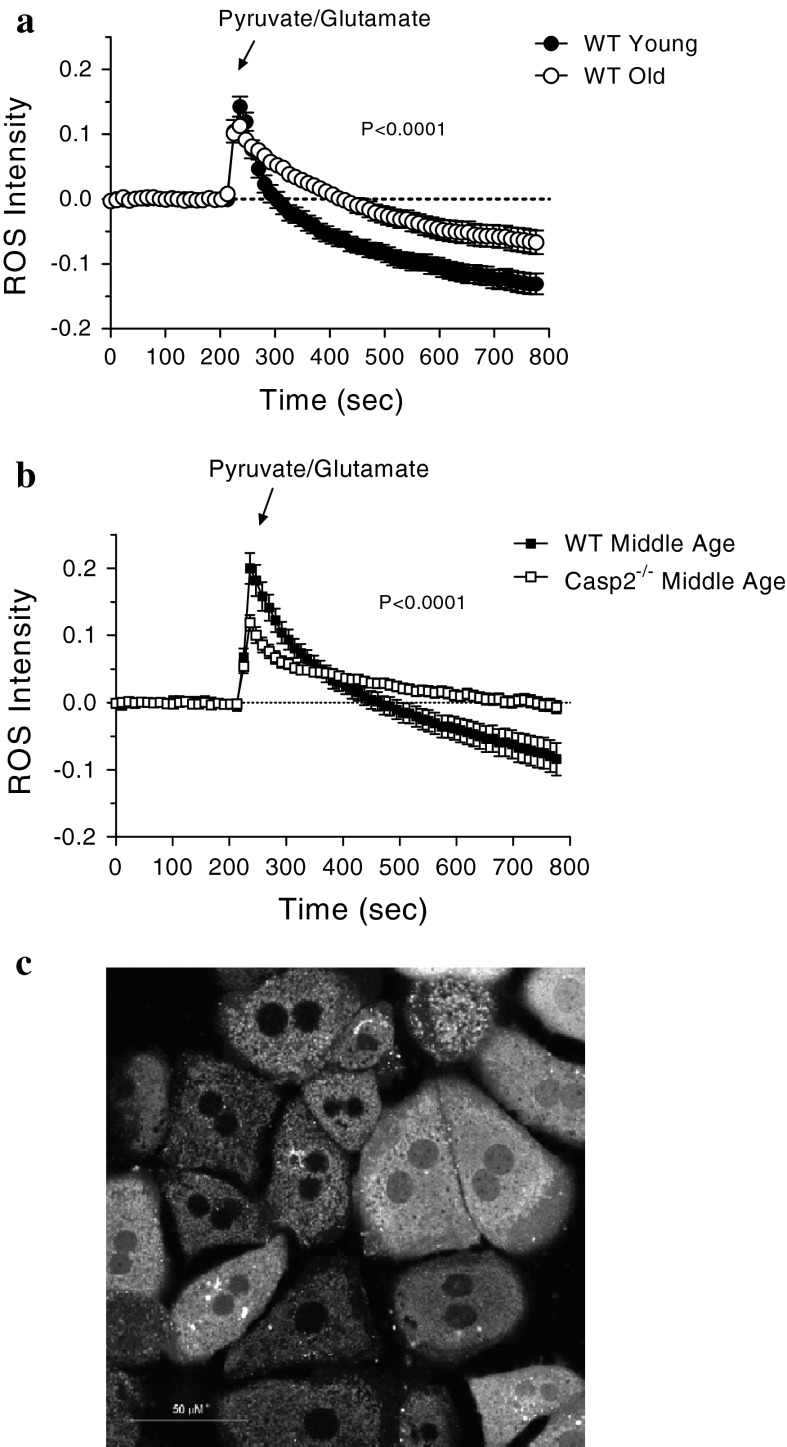
Age and casp2-dependent differences in mROS metabolism. Hepatocytes were isolated from 5 (young), 12 (middle age) and 28 (old) month old WT and 12 month *Casp2* ^−*/*−^ mice by perfusing their livers with a buffer solution containing EGTA, followed by collagenase treatment. Dispersed hepatocytes were seeded in glass-bottom well chambers and stained with MitoSOX. Cells were then placed in a confocal microscope and the production of mROS was monitored over time after the addition of a mixture of 5 mM pyruvate and glutamate. Recordings were performed for 13–15 min. **a** Mitochondrial ROS production over time in 5 and 28 month old WT mouse hepatocytes. **b** Mitochondrial ROS production over time in 12 months old *Casp2* ^−*/*−^ and WT mouse hepatocytes. Data was normalized for basal intensity. *Error bars* = ± SEM. **c** Representative image of isolated hepatocytes labeled with mitochondrial ROS probe MitoSOX. No images analyzed or visually tested showed any contamination with other type of cells

**Fig. 2:**
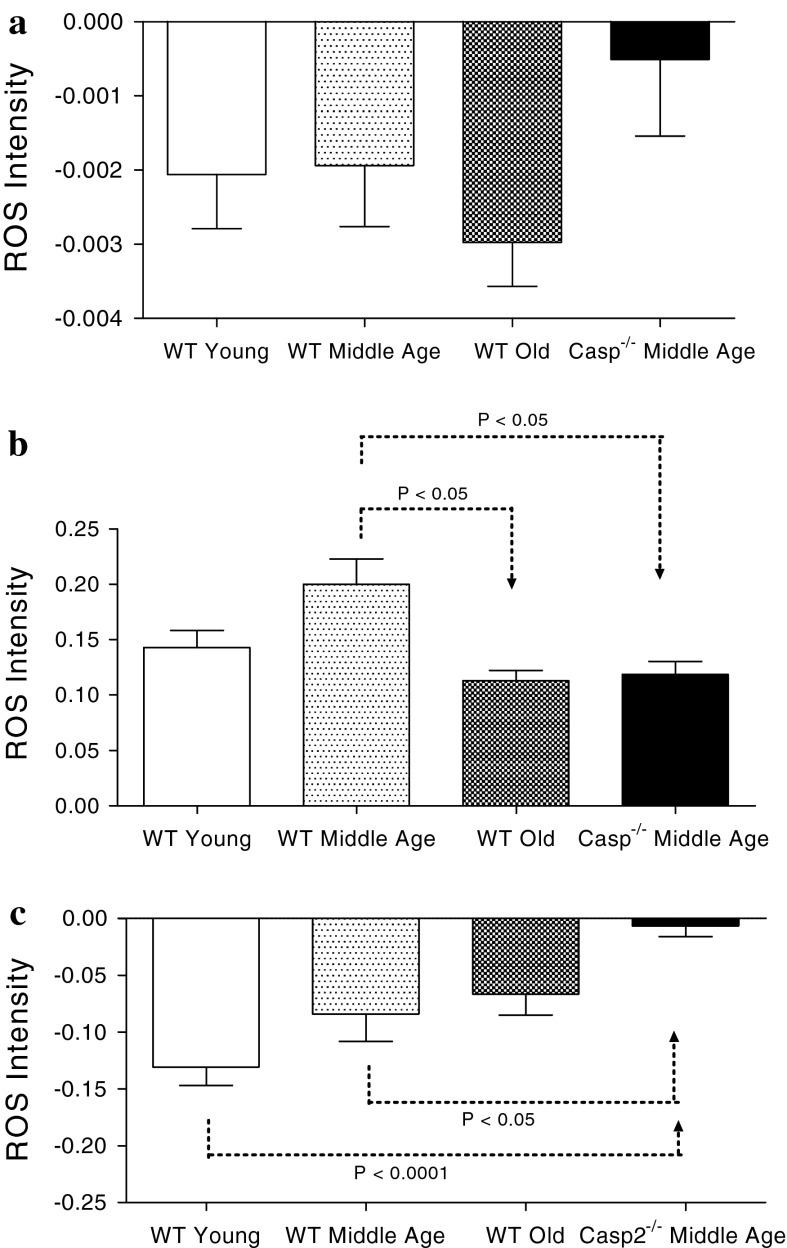
Change in mROS production/metabolism over time. Data from Fig. 1 was used to calculate differences in mROS intensities. **a** Graph shows differences at t = 0 s (baseline). **b** t = 200 s (peak after PG addition), and **c** t = 800 s (after recording began) between young, middle age, and old WT hepatocytes and middle age hepatocytes from C*asp2* ^−*/*−^ mice. Results from three experiments are shown. *Error bars* = ± SEM

In the present study, we have examined the generation of mROS as a function of age in hepatocytes isolated from WT mice and also the impact of casp2 deficiency in the generation of mROS. Liver hepatocytes were selected as a model to study the action that absence of casp2 exerts in mitochondria since these cells are enriched with this organelle. We initially investigated the endogenous generation of mROS in hepatocytes isolated from young and old WT mice upon feeding the mitochondrial electron transport chain with PG (Fig. 1a) and the mitochondrial probe MitoSOX. MitoSOX is known to selectively label mitochondrial superoxide radicals. All cells considered in these experiments showed distinct mitochondrial labeling. Hepatocytes are abundant in mitochondria due to their high metabolic activity and the basal ROS levels in hepatocytes were almost equal in both young and aged hepatocytes. There was no statistical difference observed between the dye uptake of young and aged hepatocytes. MitoSOX is reported to have a high response time so that in the time scales of measurement, it is ensured that the probe senses the free radicals in real time. Mitochondria from younger WT hepatocytes tended to produce slightly more mROS than older WT hepatocytes at the peak of PG stimulation (Figs. 1a, 2a). However, as expected, younger hepatocyte mROS neutralization kinetics was more efficient compared to the mROS kinetics of hepatocytes from older mice. Since we are interested in analyzing the role that casp2 plays in mitochondrial oxidative stress induced apoptosis, we next assayed the response of PG-induced mROS metabolism (we defined the metabolism of mROS as the amount of decrease in the MitoSOX signal at t = 200 s to t = 800 s) in *Casp2* ^−*/*−^ hepatocytes isolated from middle age mice and compared it to the response of hepatocytes obtained from age-matched WT mice (Fig. 1b). We chose middle age animals because in parallel with the present studies, our laboratory has demonstrated that liver from middle age *Casp2* ^−*/*−^ mice exhibit a general increase in protein oxidation identical to that seen in old WT mouse livers (Zhang et al. 2007). Hepatocytes from middle age *Casp2* ^−*/*−^ mice demonstrated lower maximal mROS production and decreased metabolism of mROS compared to hepatocytes from age-matched WT mice. In fact, the middle age *Casp2* ^−*/*−^ mROS levels never reached below the baseline for the duration of the measurement, while middle age WT mROS level quickly went below the baseline. It is important to note that the hepatocytes isolated and used for these experiments (Fig. 1c) were free from contamination with other cell types as seen by the presence of binucleate cells and the richness of mitochondria within each cell. This technique has been employed in our laboratory for more than 20 years (Herman et al. 1988).

The graphs in Fig. 2 demonstrate differences in mROS intensity at baseline (i.e. before PG was added, t = 200 s), peak mROS levels after PG stimulation (t = 250 s) and mROS decay at 800 s after the recording began. Changes in baseline intensity were relatively small for all ages of hepatocytes and there were no statistically significant differences in baseline mROS levels (t = 200 s; Fig. 2a; note minute scale). However, peak mROS intensities obtained after PG stimulation did show substantial and statistically significant differences between ages and casp2 content (Fig. 2b). Hepatocytes from middle age WT mice showed the greatest increase in mROS production. In comparison, hepatocytes obtained from old WT mice showed statistically significant lower PG stimulated increases in mROS levels. mROS production in hepatocytes obtained from young WT mice did not show a significant difference compared to those from WT middle age mice. Interestingly, mROS levels seen in old hepatocytes following PG stimulation were identical to those seen in PG-stimulated hepatocytes obtained from middle aged *Casp2* ^−*/*−^ mice. In addition to peak mROS production, we also examined how well hepatocytes metabolized PG-induced mROS as a function of age and in the absence of casp2 (Fig. 2c). We observed an age-dependent impact on the ability of hepatocytes to metabolize mROS. Young hepatocytes were the most efficient while the least effective were hepatocytes from old WT mice. Interestingly, the most compromised of all hepatocytes in terms of mROS metabolism were those obtained from middle-aged *Casp2* null mice.

**Fig. 3:**
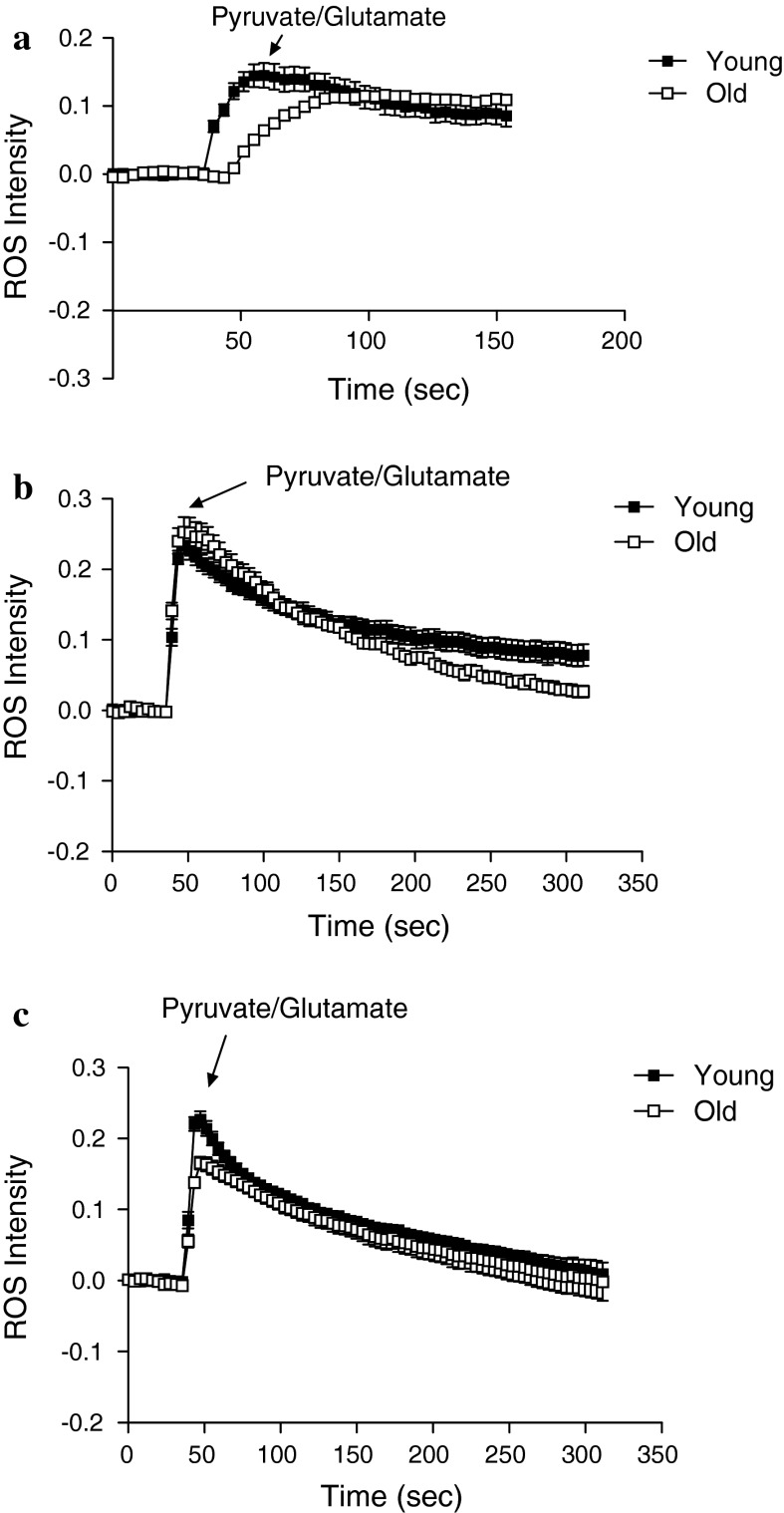
Hepatocytes from young and old WT mice show the same mROS response following exposure to rotenone. Hepatocytes were isolated from 5 and 28 month old WT mice by perfusing their livers with a buffer solution containing EGTA, followed by collagenase treatment. Dispersed hepatocytes were seeded in glass bottom well chambers, treated with 20 μM of rotenone for 30 min, and stained with MitoSOX. Cells were then placed in a confocal microscope and the production of mROS was monitored over time after the addition of a mixture of pyruvate and glutamate. Recordings were performed over a period of 13–15 min. The results from three independent experiments are shown in **a**, **b** and **c**. Data was normalized for basal intensity. *Error bars* = ± SEM

**Fig. 4:**
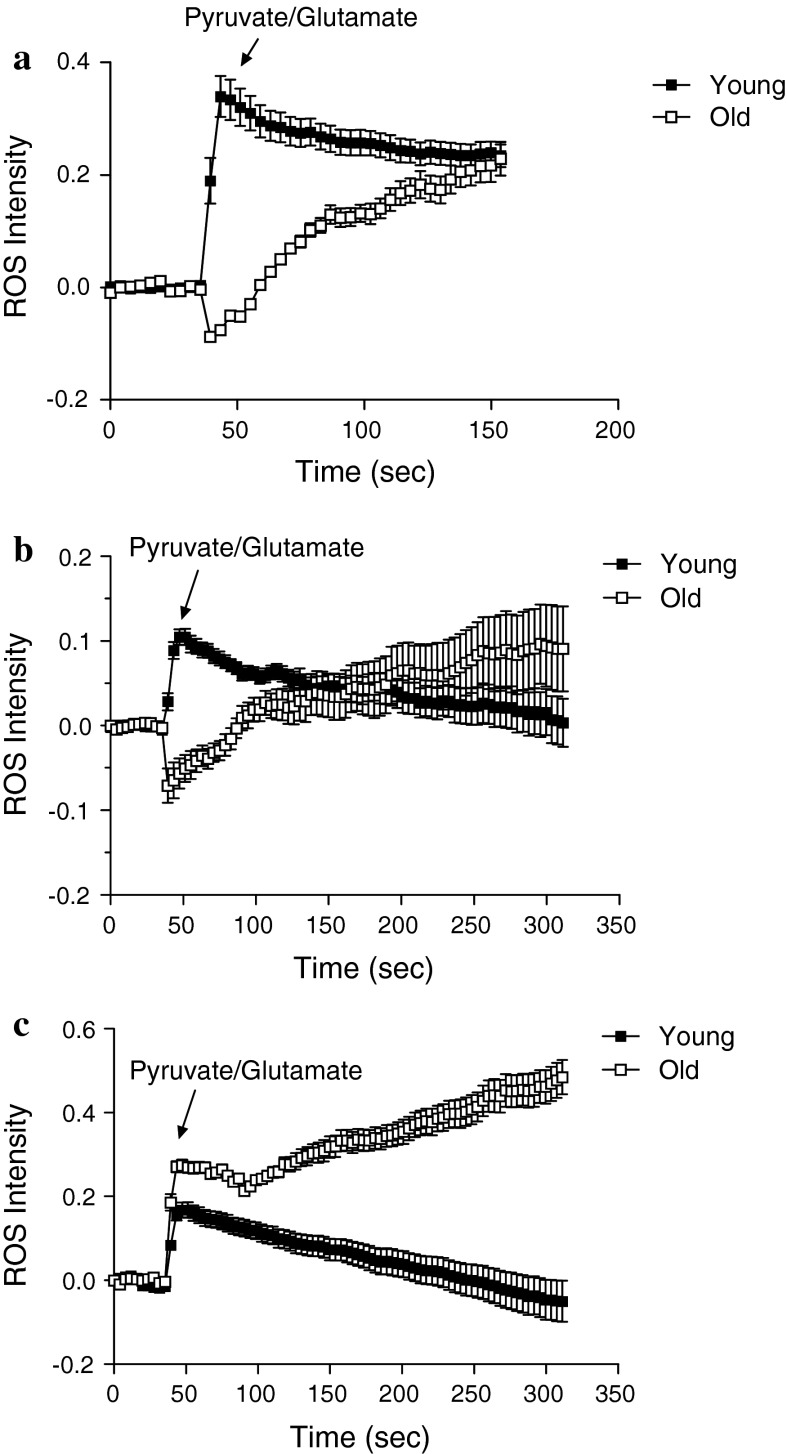
Hepatocytes from young and old WT mice show a marked difference in mROS response to antimycin A treatment as a function of time when the electron transport chain is fed with pyruvate and glutamate. Hepatocytes were isolated from 5 and 28 month old WT mice by perfusing their livers with a buffer solution containing EGTA, followed by collagenase treatment. Dispersed hepatocytes were seeded in glass bottom well chambers, treated with 20 μM of antimycin A for 30 min, and stained with MitoSOX. Cells were then placed in a confocal microscope and the production of ROS from the mitochondria was monitored over time after the addition of a mixture of pyruvate and glutamate. Recordings were performed for 13–15 min. The results from three independent experiments are shown in **a**, **b** and **c**. Data was normalized for basal intensity. *Error bars* = ± SEM

**Fig. 5:**
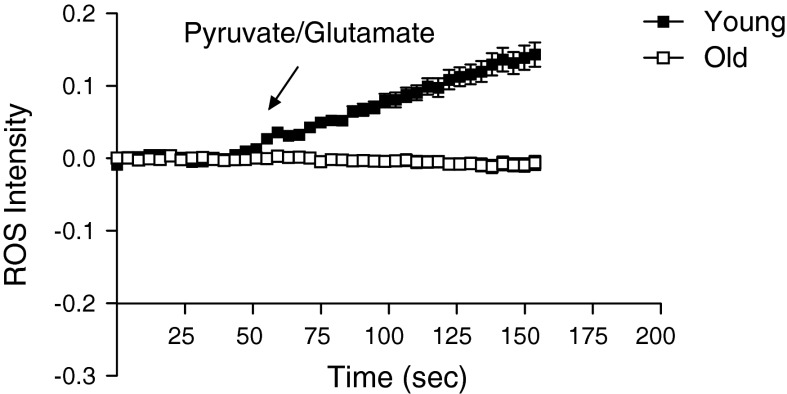
Hepatocytes from young and old WT mice show a marked difference in mROS response to a mixture of rotenone plus antimycin A treatment as a function of time when the electron transport chain is fed with pyruvate and glutamate. Hepatocytes were isolated from 5 and 28 month old WT mice by perfusing their livers with a buffer solution containing EGTA, followed by collagenase treatment. Dispersed hepatocytes were seeded in glass bottom well chambers, treated with a mixture of 20 μM of rotenone plus antimycin A for 30 min, and stained with MitoSOX. Cells were then placed in a confocal microscope and the production of ROS from the mitochondria was monitored over time after the addition of a mixture of pyruvate and glutamate. Recordings were performed for 13–15 min. The results from three independent experiments are shown

Next we sought to dissect the genesis of the changes in mROS that we observed. Therefore, we investigated mROS metabolism in hepatocytes from young and old WT mice following inhibition of complex I and III by rotenone (Fig. 3a, b, c) or antimycin A (Fig. 4a, b, c) respectively, in a time dependent manner. We used the same concentrations of rotenone and antimycin A, and incubation times to pre-treat hepatocytes, and then created a burst of mitochondrial respiration by adding a mixture of PG while scanning images of live cells to detect mROS previously stained with MitoSox Red. Figures 3 and 4 show graphs of three independent experiments that we present separated to avoid masking the results by the large standard error that they generate, but to show the trend in treatments of rotenone and antimycin A. mROS kinetics of rotenone treated hepatocytes failed to demonstrate any differences in terms of the maximum burst of mROS or the kinetics of mROS metabolism between hepatocytes from young versus old WT mice (Fig. 3). On the other hand, pre-treatment of hepatocytes with antimycin A resulted in a striking and unexpected difference in the mROS kinetics curves obtained from hepatocytes isolated from young versus old WT mice (Fig. 4). While the response of the hepatocytes from young mice to antimycin A treatment was similar to that seen in rotenone treated hepatocytes (i.e. an initial upward tick and subsequent downward slope), hepatocytes from old mice showed the opposite outcome. Addition of PG in antimycin A pre-treated hepatocytes from old mice resulted in a sudden decrease in the generation of mROS that slowly and progressively returned towards pretreatment levels as a function of time. We also pre-treated hepatocytes isolated from WT mice with a mixture of both rotenone and antimycin A (Fig. 5). After addition of PG, no initial response was seen in either WT young or WT old. However, young WT hepatocytes progressively developed mROS in an upward linear fashion.

Taken together our results indicate that: (1) there is a difference between the way hepatocytes from young and old mice respond to dysfunction of the mitochondrial electron transport chain and subsequent production and neutralization of mROS; (2) this difference is most probably due to age dependent dysfunction of complex III; and (3) absence of casp2 in hepatocytes obtained from middle age mice respond in a similar way as hepatocytes obtained from old WT mice in terms of PG-induced mROS metabolism.

## Discussion

Our previous studies have shown a progressive increase in the activity of caspases that mediate apoptosis induced through the mitochondrial pathway as liver ages (Zhang et al. 2002). This suggests a strong contribution of the intrinsic pathway of apoptosis to liver aging. It is generally agreed that oxidative stress, which is a potent inducer of apoptosis, increases with age. The speculation that mitochondrial oxidative stress may underlie age-associated increases in apoptosis in the liver is supported by the observation that liver hepatocyte apoptosis is higher in old mice with half the amount of MnSOD than in age-matched WT mice (Kokoszka et al. 2001). These results validate previous studies that showed increase numbers of TUNEL positive hepatocytes in livers of aged Fisher 44 rats (Higami et al. 1997). In addition, we have also shown that hepatocytes isolated from old rats are more sensitive to oxidant-induced cell death than hepatocytes isolated from young rats (Zhang et al. 2002).

Casp2 has been implicated in apoptotic and non-apoptotic processes such as cell cycle regulation, tumor suppression and aging (Bouchier-Hayes and Green 2012 for review). Casp2 has also been identified as a central player in the mitochondrial pathway of apoptosis (Guo et al. 2002; Boatright et al. 2003; Enoksson 2004). Furthermore, casp2 has been reported to be localized in the mitochondrial compartment (Susin et al. 1999, Deaciuc et al. 2004, Cheung et al. 2006), although the localization of casp2 in mitochondria is still under debate (Mancini et al. 2000, O’Reilly et al. 2002, van Loo et al. 2002). Our laboratory, as well as other groups, has suggested that casp2 mediates apoptosis induced by the lipid peroxidant tert-butyl hydroperoxide (tBOOH) (Amoroso et al. 2002, Zhang et al. 2002). Mitochondria are a major site of ROS generation and excess ROS triggers apoptosis mediated by caspases. Consequently, the role of casp2 as an initiator of mitochondrial apoptosis is now commonly accepted.

To examine the potential role of casp2 in the relationship between aging and mitochondrial oxidative stress, we examined the implications of removal of casp2 on hepatocyte mROS metabolism. These studies have been performed in male mice for three reasons: (1) All our previously published data on casp2 deficient mice biology has been obtained using male mice; and (2) hepatocytes used in these experiments were cultured in vitro with specific media, thus removing any potential impact of gender in the results. Hepatocytes are cells with a high degree of metabolic activity and are greatly enriched in mitochondria, which in turn leads to production of high levels of ROS. We examined ROS production from mitochondria in hepatocytes isolated from young (5 months), middle age (12 months) and old (25 months) WT mice. Our data demonstrated that hepatocytes of old mice do not produce as much initial mROS as hepatocytes from young mice when mitochondrial respiration is accelerated. In addition, the kinetics of mROS neutralization were not as efficient in hepatocytes isolated from old mice compared to that observed in hepatocytes isolated from young mice. These results indicate that older hepatocytes lack the metabolic efficiency found in younger hepatocytes.

Inhibiting certain complexes of the electron transport chain results in an increase in mROS production. Thus, we examined the level of ROS production following metabolic stimulation in the presence and absence of inhibitors of the electron transport chain. The two main sites responsible for ROS production following inhibition are complex I and complex III. We used rotenone and antimycin A to inhibit these electron transport chain complexes, respectively. Our results demonstrate that while there is no difference in ROS generation between young and old hepatocytes following complex I inhibition, hepatocytes from old WT mice respond very differently to inhibition of complex III in the mitochondrial electron transport chain and subsequent generation and neutralization of mROS than hepatocytes from young mice. However, we did not detect any differences when complex I was inhibited among hepatocytes of either age. These results lead us to hypothesize that older hepatocytes are less capable of handling the same or greater levels of mROS produced during inhibition or dysfunction mainly of complex III. Following treatment with both rotenone and antimycin A, the initial rapid increase in mROS production observed after metabolic stimulation was completely inhibited. Subsequently, a steady increase in mROS production was observed only in young hepatocytes. These results suggest that first, aging is associated with differences in mROS metabolism and second, that aging affects complex III activity preferentially with respect to mitochondrial oxidative stress. We also show that hepatocytes from middle age *Casp2* ^−*/*−^ mice display a greater difficulty in neutralizing mROS than hepatocytes from their age-matched WT counterparts, and in fact resemble the characteristics of mROS neutralization found in hepatocytes obtained from old WT mice. These data implicate casp2 in the preferential metabolism of mROS generated from complex III. Recent evidence suggests that when complex III is inhibited, complex II may be a source of ROS (Quinlan et al. 2012). We examined mROS production in young and old WT hepatocytes containing non-functional complex I and III (Fig. 5). Our results indicate that complex II is not a source of ROS and it is in agreement with previous findings (Chen et al. 2003), using glutamate, but not succinate as a substrate. Since the equivalency of mROS metabolism in middle aged *Casp2* ^−*/*−^ mice is comparable to old WT mice, our data suggests that casp2 is an important player in the aging process resulting from oxidative stress.

At the present time, the mechanisms by which casp2 may sense and respond to complex III generated ROS or its targets are unknown and are the focus of ongoing studies. However, recent findings suggest a number of feasible mechanisms by which casp2 may regulate cellular ROS levels and responses. Higher levels of ROS can damage enzymes responsible for production of NADPH and Calmodulin dependent kinase II (CaMK II) (Erickson et al. 2008). In addition, scavenging ROS or repairing oxidized macromolecules consume NADPH. These changes can potentially impair the function of CaMK II. Because CaMK II phosphorylates the cysteine apoptotic protease, procaspase-2, at Ser135 and inhibits its activation (Nutt et al. 2005), it is possible that partial loss of CaMK II function reduces the threshold of oxidative stress required for the activation of casp2, resulting in apoptosis. NADPH, a co-factor of several anti-oxidant enzymes such as glutathione reductase and thioredoxin reductase, enhances CaMK II-mediated inhibition of casp2 (Lee et al. 2001). Because NADPH is a critical molecule in redox regulation, casp2 activity may be ultimately regulated by cellular redox. Indeed, casp2 is necessary for mitochondrial oxidative stress-induced apoptosis because its absence protects cells treated with mitochondrial complex I inhibitors such as rotenone from apoptosis (Lopez-Cruzan et al. 2005, Tiwari et al. 2011). In addition, we find casp2 activity to be increased prior to other caspases in MnSOD-partially-deficient mice that have enhanced mitochondrial oxidant stress (data not shown). Because it is the most conserved caspases (shares >90 % homology with human casp2), we aligned the protein sequence of casp2 from several species and found that casp2 contains 17 cysteine residues conserved across. Thus, casp2 is the highest cysteine containing caspase out of all the known caspases. Cysteine residues are known to regulate protein function via oxidation and reduction of disulfide bonds as a function of the redox environment. One of these 17 cysteine residues is involved in the dimerization of casp2 monomers via formation of a disulfide bond that stabilizes the molecule (Schweizer et al. 2003), but is apparently not required for casp2 activation. Four of the remaining 16 cysteines are aligned in the dimer in a conformation accessible to sense the redox environment, but do not form disulfide bridges under physiological conditions. We hypothesize that one or more of these 4 cysteines might serve as sensors of mitochondrial oxidative stress, potentially via oxidation.

In earlier studies published by us employing a unique combination of spatially resolved single-cell chemical kinetics, scaling analysis, and biochemical assays, we observed that young liver cells manifest nonlinear dynamics for efficiently regulating ROS generation/removal machinery, and that these regulatory correlations in free radical dynamics are diminished in aged cells, suggesting that the aging process modulates chemical dynamics (complexity) in liver cell energy metabolism (Ramanujan and Herman 2007). We speculated that these differences arise from nonlinear network interactions among glycolysis, gluconeogenesis, and mitochondrial electron transport chain. Pyruvate dehydrogenase is the key enzyme that converts glycolytic product pyruvate to acetyl-CoA as an input to mitochondrial pathway, and it is known that pyruvate dehydrogenase is inhibited by excess NADH/acetyl-CoA (product inhibition). If the mitochondrial pathway is defective in aged cells, unmetabolized pyruvate will be converted back to glucose by gluconeogenesis, which then will initiate the glycolytic pathway. It has been reported earlier that aging is associated with ROS-induced chronic dysfunction of mitochondrial respiratory chain, either at site I or III, and that mitochondria isolated from aged animals show reduced sensitivity to complex I inhibitor (Fosslien 2001). Similar trends were observed in these studies, suggesting that aging in liver is accompanied by a dysfunctional mitochondrial network that can be due to structural or functional respiratory defects, or both. These findings are consistent with the data reported here, that defects in complex III accompany the aging process and loss of casp2.

Lastly, a recent publication (Shalini et al. 2012), that repeats and recapitulates our previous finding in bone, livers, mouse embryonic fibroblasts and hepatocytes from *Casp2* ^−*/*−^ mice (Lopez-Cruzan et al. 2005, Zhang et al. 2007, Tiwari et al. 2011) showed that old *Casp2* ^−*/*−^ mice have increased cellular levels of oxidized proteins, lipid peroxides and DNA damage. Fibroblasts and neurons from *Casp2* null mice generate higher levels of endogenous ROS that is associated with decreased levels of antioxidant enzymes (Tiwari et al. 2011). Casp2 was found to upregulate anti-oxidant protein expression by activating the transcription factor FoxO family (Shalini et al. 2012). In particular, FoxO3 is responsible for expression of catalase, MnSOD, and sestrins genes; the later which activates the peroxiredoxin family of H_2_O_2_ detoxifiers. In the absence of casp2, these antioxidants are decreased, and in the case of MnSOD, it might explain the increased mROS production seen in older WT and middle age casp2 null hepatocytes. Taken together, these observations demonstrate strong mechanistic links between casp2, oxidative stress and aging.
